# A day of shame

**DOI:** 10.12669/pjms.40.10.10622

**Published:** 2024-11

**Authors:** Shabih H. Zaidi

“This is a day of shame “announced the British PM Rishi Sunak in the parliament on 21 May 24, in the Houses of Parliament. The background of this statement is the story called the ‘contaminated blood scandal’.^1^ A large number of people (c30,000) were infected with Hepatitis-C and HIV, after receiving contaminated blood or blood products. The NHS used them throughout the 1970s-80s. It is believed that c3000 have since succumbed and many continue to live a life of morbidity, health issues and stigma.

The British media has recently reported many unhappy stories. One of them relates to the Lord Mayor Treloar College in Hampshire where boys were used as “objects for research”, worse than the chimpanzees, as the inquiry concluded.^2^ ‘Only 30 of 122 pupils with haemophilia at the school in the 1970s and 80s were still alive, its final report added’. The college said it was “devastated” that former pupils were affected.^3^ The government has apologized not just for the present conservative government, but also on behalf of previous governments and the NHS. It has come as a sledgehammer hitting the head of crumbling NHS. The government will pay out 10 Billion pounds to hundreds of victims of the horrendous tragedy.

Experimental drugs, vaccines, blood products, procedures have been used for ever in the fields of medicine and health care. Historically we can go back to the days of ancient civilisation of the Assyrians, Egyptians, Greeks and Romans. Even great Galen may have carried out such experiments on the slaves, which were an easy fodder for trials and triumphs. ‘Slaves were not alone. Many poor souls were subjected to medical testing. In Europe and its American colonies, drug trials tended to over-select subjects from the poor and wards of the state, such as prisoners, hospital patients and orphans’.^4^

The infamous Tuskegee trial is worth mentioning here, as not many people know much about it. The U.S. Public Health Service (USPHS) Untreated Syphilis Study at Tuskegee was conducted between 1932 and 1972 to observe the natural history of untreated syphilis. As part of the study, ‘researchers did not collect informed consent from participants and they did not offer treatment, even after it was widely available.^5^ President Clinton had to televise his unconditional apology to the African - American victims during his last days of presidency.

When I was a postgraduate student in London, a famous professor of Physiology at the Royal College of Surgeons in Lincoln’s inn told us about an ice breaking whistleblowing story that had just unfolded. It became a milestone over time. It was Maurice Papworth, who published a book in 1967, exposing the atrocities faced by human beings involved in experimental research. He was the first physician to use the word Human Guinea pigs, echoed today on the British television and print media.^6^ No doubt science cannot advance without research. But involving human beings for research deserve stringent application of rules and regulations formulated by competent authorities over the years.

It is time to revisit the stories of Nazi experiments in various prisoner camps during the Second World War which were exposed at the Nuremberg trials. The world was aghast and authorities gathered swiftly to develop and design the famous Nuremberg code of human experimentation. It comprised 10 basic rules, focusing on the fundamental principle of human dignity. 7

This code recognizes that doctors should avoid actions that injure human patients. The principles established by this code for medical practice have been well documented. The Helsinki declaration came into force in 1964, which manifested the rules of research. It has witnessed numerous modifications over the years, and one suspects may continue to do so in the future.^8^

A few years later came the famous Belmont report, which is not so well known or instantly recognized in scientific circles.^9^ It comprises three core principles which are : respect for persons, Beneficence, and justice. They are acknowledged by all ethicists universally. Beauchamp and Childress identified Four principles namely Autonomy, Beneficence, Non maleficence and Justice in their famous book. Beneficence however takes precedence over autonomy in most Eastern cultures, where collective or familial consent is the norm.

Malpractice is a common phenomenon in health care. Sonia Shah published a book in 2006, which highlighted the role of the pharmaceutical industry, in the abuse of authority, use of bribes of large sums of money, in conducting drug trials and human experimentation. It is an eye opener to read.^10^ The process continues without remorse.

The pandemic of the COVID-19 has unfolded many ethical challenges. The world was totally unprepared for a pandemic. It was therefore caught in a state of Lilliputian slumber. Who was responsible and who should ensure that the same would not happen again, is a matter of debate another time. World health authorities failed, states crumbled and politicians lied. The outcome of the disaster caused by neglect and lies is simply unprecedented.

January 2020, saw many health professionals perish. The first physician to succumb was my class fellow and a dear friend. The family still can’t believe what hit them. The peril of COVID unfolded gradually. The first few Muslim bodies probably lacked proper burial, as no one wanted to touch them let alone perform a ritual bath.

Death overwhelmed everything. Hundreds of thousands were dying, so many ethical principles had to be compromised. Utilitarianism took the lead as scarcity of ventilators and oxygen, hospital beds and services required to be shared with the needy people prioritizing the medical need rather than age or QALYs, so affectionately used by health economists in routine matters.

As people lay dying, frantic search for vaccines was made. The first vaccine arrived in a few months after the onset of the pandemic. The question cropped up later, whether enough reliable data was available to justify their use in human beings? It takes 10 years to develop a vaccine, which was manufactured in just 10 months. It has been a matter of concern.

Soon we noted the emergence of a group of people called antivexers, as the famous sports personalities, politicians and health professionals took the lead to condemn vaccines. Time would prove that they were following in the footsteps of one Andrew Wakefield, a disgraced pediatrician who had raged a war against mandatory vaccination. He was struck off the medical register for his involvement in *The Lancet* MMR autism fraud, a 1998 study that fraudulently claimed a link between the measles, mumps, and rubella (MMR) vaccine and autism. He has subsequently become known for anti-vaccination activism.^11^,^12^

Healthy volunteers are often engaged in clinical trials. Such ads are commonly seen in the national newspapers seeking such volunteers. Once in a while such a trial may go wrong. The notable Northwick park hospital episode of a drug trial going wrong^13^ while a trial was carried out on TGN1412, CD28-SuperMAB, and TAB08) In what became known as the Elephant Man trial, six healthy young men were treated for organ failure after experiencing a serious reaction within hours of taking the drug.

The 4th round of COVID vaccination has resulted in many complaints of side effects varying from mild skin rash to severe anaphylaxis, mind fogging, blood clots, and even a case of fatality. It has resulted in withdrawal of AstraZeneca vaccine, though claimed to be a policy matter.^14^

The spring of 2024 saw a rising number of whooping cough and measles in the U.K., as many mothers had avoided vaccination of their children with MMR. Similar stories are emerging from many other countries. Pakistan, regrettably, remains on the radar for the on going battle against polio. Many local tribesmen, even religious scholars, had previously opposed immunization in the northern territories.

This recent historical speech by the prime minister in the mother of all parliaments, raises many questions in the field of research involving human beings.

1. What should be the new criteria of human experimentation?
2. Who should monitor it.
3. Who should be held responsible for the adversities.
4. How should the affected be compensated?
5. And a major question of the unethical practices carried out in the developing countries day in and day out. Lord save us all.
